# Structural and Functional Insights into the Role of Guard Cell Ion Channels in Abiotic Stress-Induced Stomatal Closure

**DOI:** 10.3390/plants10122774

**Published:** 2021-12-15

**Authors:** Hamdy Kashtoh, Kwang-Hyun Baek

**Affiliations:** Department of Biotechnology, Yeungnam University, Gyeongsan 38541, Gyeongbuk, Korea; hamdy_kashtoh@ynu.ac.kr

**Keywords:** guard cell, abiotic stress, KAT1, SLAC/SLAH, abscisic acid signaling, kinases

## Abstract

A stomatal pore is formed by a pair of specialized guard cells and serves as a major gateway for water transpiration and atmospheric CO_2_ influx for photosynthesis in plants. These pores must be tightly controlled, as inadequate CO_2_ intake and excessive water loss are devastating for plants. When the plants are exposed to extreme weather conditions such as high CO_2_ levels, O_3_, low air humidity, and drought, the turgor pressure of the guard cells exhibits an appropriate response against these stresses, which leads to stomatal closure. This phenomenon involves a complex network of ion channels and their regulation. It is well-established that the turgor pressure of guard cells is regulated by ions transportation across the membrane, such as anions and potassium ions. In this review, the guard cell ion channels are discussed, highlighting the structure and functions of key ion channels; the SLAC1 anion channel and KAT1 potassium channel, and their regulatory components, emphasizing their significance in guard cell response to various stimuli.

## 1. Introduction

Plants must adapt to different environmental challenges such as drought, elevated CO_2_ and O_3_, and pathogen attacks to survive. The guard cell plays an essential role in plants when adapting to such environmental stimuli, which are on the rise due to global warming. It is comprised of two kidney-shaped cells forming a stomatal pore in the plant leaf epidermis and is responsible for gaseous exchange between the plants and the surrounding environment [1,2,3,4,5]. Stomata opening is induced by light, allowing a CO_2_ influx during photosynthesis in the leaves, whereas stomata pore closure occurs when plants are exposed to water-deprived conditions to keep the water balance in plants maintained. Stomata movements are achieved via ion fluxes to control guard cell turgor pressure in response to environmental stimuli with a highly sophisticated signaling network that controls cation and anion channels located in the PM (plasma membrane) and tonoplast of the guard cell and is responsible for tuning the stomatal movement. Light-induced stomatal opening is governed by K^+^_in_ (inward-rectifying potassium) channels present in the PM of the guard cells, such as KAT1, KAT2, AKT1, and AKT2. In *Arabidopsis thaliana*, KAT1 and KAT2 channel activity represents a major contributor to a potassium influx in the guard cell [6,7,8,9,10,11,12,13]. On the other hand, stomatal closure requires a coordinated efflux of anions and potassium ions from the guard cell via anion channels such as S-type slow activated anion channels (S-type), R-type rapidly activated anion channels (R-type), and K^+^_out_ outward-rectifying potassium (K^+^_out_) channels [14,15,16,17,18,19,20,21,22,23,24,25,26]. It has been proposed that the activation of the S-type anion channel SLAC1 in the guard cells is a key event leading to the closing of stomata pores [17,18,27]. Abscisic acid (ABA) regulates the cellular activities that coordinate anion effluxes, inhibits K^+^_in_ channels, and activates K^+^_out_ channels in a signaling pathway mediated by ABA receptors, phosphatases/kinases, and ion channels. Upon environmental stimuli such as drought, ABA production in the guard cell increases and elevates cytosolic calcium concentrations, initiating a cascade of signaling events, thereby leading to ion effluxes outside the guard cell, reducing the turgor pressure and stomata closure which occurs to maintain water balance in plants [28,29,30,31]. There are several intertwined signaling pathways in the guard cell that regulate ion channel activity and modulate stomatal movements in response to environmental stimuli. For instance, under abiotic stress, ABA and H_2_O_2_ (reactive oxygen species (ROS)) levels increase which triggers an elevated cytosolic calcium concentration that in turn activates the calcium protein kinases which regulates ion channel activities and tolerates such abiotic stress [32,33,34]. Several other signaling cascades that regulate stomatal movements are generated in response to stress, such as plant hormones brassinosteroid (BR) signaling which helps plants tolerate stress environments [35,36,37]. Our knowledge about the functions and regulation of the ion channels in stomatal movement comes from electrophysiological studies conducted on single guard cells and the analysis of the effects of the ion channel mutants on the stomatal operation. Slow anion channel associated-1 (SLAC1) was discovered during the genetic screening for O_3_ or CO_2_ sensitivity in *Arabidopsis*. When the SLAC1 channel was mutated, the resulting plants no longer responded to O_3_ stress and elevated CO_2_ whereas a slow modest response was observed when light and air humidity were changed [17,18]. Integration of these techniques and proteomics helped elucidate signaling mechanisms contributing to guard cell movement. Insights into these mechanisms will help breed plant crops with better water tolerance. In this review, we highlight the recent progress in the signaling networks that coordinates the activation and inhibition of ion fluxes of potassium and anions such as chloride, nitrate, and malate during stomatal closure. Moreover, the structure and function of the key channels SLAC1 and KAT1 that are involved in guard cell stomatal movement are discussed.

## 2. Guard Cell Potassium Channels

There are fifteen potassium channels identified in *Arabidopsis thaliana*; nine Shaker channels and six TPKs two-pore K^+^ channels (TPKs) [38,39]. Shaker family potassium channels are best characterized and they mediate the potassium fluxes at the plasma membrane. The members of the shaker family can be classified into two categories: (i) inward rectifying channels, which include KAT1, KAT2, AKT1, AKT2, AKT6, SPIK, and AtKC1, and (ii) outward rectifying channels that include GORK and SKOR [13,40,41]. Six of the shaker family members are expressed in the guard cells and have a significant role in the stomatal opening (KAT1, KAT2, AKT1, AKT2, and AtKC1) and closing (GORK) [9,13,42]. A complex array of signaling cascades will lead to the activation of K^+^_in_ channels. Light and other pathways such as binding of 14-3-3 protein, Ca^2+^ elevation, K^+^ influx, and ABA-induced H_2_O_2_ activity mediate stomatal movement by regulating PM H^+^-ATPase activity [43,44,45,46,47,48,49]. The PM H^+^-ATPase receives the blue light-activated signal, which causes phosphorylation of the H^+^-ATPase C-terminus [44]. When activated by blue light and Fusicoccin, the 14-3-3 protein binds to the PM H^+^-ATPase, which raises the negative electrical potential gradient inside the PM and drives K^+^_in_ channels [44,47]. As a result, the uptake of potassium ions leads to an increase in the turgor pressure of the guard cell, ensuing stomatal opening [12,50,51]. On the other hand, when K^+^_out_ channels are activated, potassium ions and anions are released along with water effluents. Subsequently, guard cell turgor pressure decreases leading to stomatal closure [52,53]. 

Shaker channels are composed of four alpha subunits that can be assembled from identical shaker genes, forming homomeric channels, or from different shaker genes, generating heteromeric channels, which is more preferable in *Arabidopsis thaliana* to increase functional diversity [54,55,56]. The tetramers are orderly positioned surrounding a central pore, which is selective for potassium ions. Each alpha subunit is comprised of six transmembrane (TM) segments forming the hydrophobic core with a potassium selectivity filter and cytosolic N-and C-termini. The pore loop domain (P domain) membrane links the fifth and the sixth TM segments forming the walls of the channel pore. The P domain comprises highly conserved residues (threonine-valine/threonine-glycine-tyrosine-glycine) at the narrowest area of the pore and serves as a selectivity filter. Positively charged amino acids (lysine or arginine) are present at the fourth TM segment, which controls the shaker channel gating by voltage; the changes in TM electrical potential cause this segment to move within the membrane, resulting in conformational changes in the channel and consequently leading to the opening or closure of the channel pore. Shaker channels have a rather large C-terminus that contains multiple domains comprising a cyclic-nucleotide binding site in addition to an ankyrin repeat domain responsible for interaction with regulatory proteins, and an acidic (KHA) C-terminal end [11,57,58,59,60,61].

**Figure 1:**
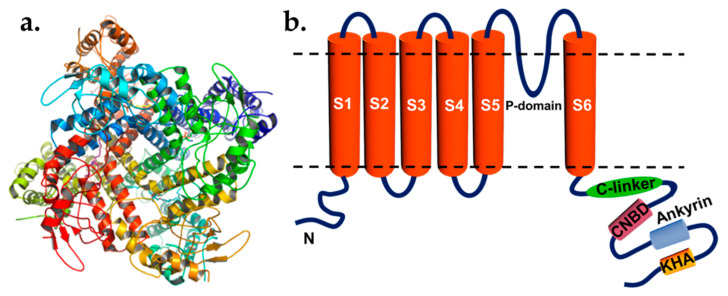
(**a**) Ribbon diagram of the structure of the *Arabidopsis thaliana* K^+^_in_ KAT1 tetramer (top view). (**b**) Topology of KAT1 subunit based on its structural model. (**a**) Was adopted from the structure, PDB entry code: 7CAL [65], and generated using PyMol [66].

KAT1 is considered a major K^+^_in_ channel in the guard cell that has a dominant role in stomatal opening [7,9,62]. Interestingly, a recent publication reports that the Shaker-type channel KDM1 in *Dionaea muscipula*, which represents an ortholog of the *Arabidopsis thaliana* KAT1, was not expressed in *D. muscipula* guard cells. It thus implies that stomatal opening in *D. muscipula* is independent of KDM1, though further experiments are needed to prove this concept [63]. KAT1 and KAT2 are expressed in the guard cell with a higher level than other K^+^_in_ such as AKT1 and AKT2. Although KAT1 is considered a dominant K^+^_in_ channel in the guard cell, the stomatal opening is still observed when it is knocked out and that may be due to KAT2, AKT1, and AKT2 subunits that can compensate for KAT1 loss [6,8,9]. Recently, the structure of the *Arabidopsis* KAT1 potassium channel was revealed. The KAT1 structure comprises a tetramer channel where each protomer contains four TMs (helices S1-4) known as a voltage sensor domain (VSD) and two TMs (helices S5, S6) of the pore domain (helices S5, S6). The helix S6 bends sharply at its C terminus, generating the C-linker helix-turn-helix motif followed by an intracellular polypeptide chain, generating the cyclic nucleotide-binding domain (CNBD). The study shows that an inbound movement of the S4 sensor helix of about 5–7 Å can cause an interaction between the sensor segment and the C-linker, resulting in changes in the conformation of the C-linker and eventually opening the activation gate formed by the S6 via a direct coupling mechanism (Figure 1) [64,65].

## 3. Guard Cell Anion Channels

**Figure 2:**
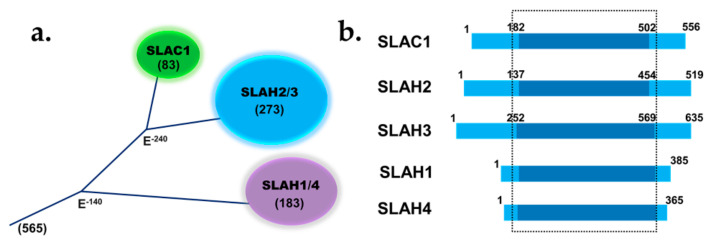
(**a**) The phylogenetic tree of the SLAC1 protein family. (**b**) SLAC1 family diagram based on sequence alignment shows SLAH1 and SLAH4 have a short N-terminus and a shorter C-terminus compared with the other family members. The N and C-termini are colored blue and the TM domains are colored navy blue.

In the 1980s, patch-clamp studies showed that anion channels in the guard cell can be distinguished into two types: Slow (S)-type and rapid (R)-type [67]. As the name implies, the R-type channel activates swiftly by depolarization, while the S-type channel activates slowly in a voltage-dependent manner [67,68,69,70]. Almost three decades later, a mutational analysis on *Arabidopsis* revealed that an ozone-sensitive mutant, with high stomatal conductance, lacks the activity of S-type guard cell anion channels by calcium or ABA. This mutant was therefore called SLAC1, and the SLAC1 gene encodes an S-type anion channel in the guard cell [17,18]. In *Arabidopsis*, SLAC1 is considered the founder of a gene family that contains SLAC1 and four of its homologue genes (SLAH1-4). The phylogenetic tree of the SLAC1 family has two branches, with SLAC1, SLAH2, and SLAH3 on one branch and SLAH1 and SLAH4 on the other [71] (Figure 2a). SLAC1, SLAH2, and SLAH 3 have cytoplasmic N and C-termini, while SLAH1 and SLAH4 have a very short N-terminus and a shorter C-terminus compared with the other family members (Figure 2b). SLAC1 and its homologue SLAH3 are expressed in the guard cell and are essential for stomatal closure [24,72]. A recent study using the optogenetic approach showed that the activation of the light-gated anion channel rhodopsin 1 (GtACR1) in tobacco guard cells was sufficient to close stomata [27]. This finding provides strong evidence that anion channels activation, such as SLAC1, is sufficient for stomatal closure. Although SLAC1 and SLAH3 activity led to stomatal closure, SLAH3 possesses some unique features. For instance, SLAC1 is permeable to nitrate and chloride, though, SLAH3 chloride/nitrate permeability is low [24]. However, SLAH3 chloride/nitrate permeability increased when SLAH1/SLAH3 are co-expressed in *Xenopus oocytes* which enabled the formation of a heterodimer [73]. Furthermore, unlike SLAC1, SLAH3 needed a rise in extracellular nitrate concentrations for complete channel activation [24,73,74]. Nevertheless, both anion channels share activation by most of the calcium-dependent kinases [24,74,75]. A recent study showed that under microbial immune response in plants, the activation of SLAH3 by a receptor-like cytoplasmic kinase PBL27 contributes to stomatal closure [76]. Another interesting study showed that in *Arabidopsis thaliana* roots, the SLAH3 anion channel is activated under flooding stress due to the acidification of the cytosolic pH and thus it was proposed that SLAH3 might act as a pH sensor to start the flooding stress complex signal response via membrane depolarization [77]. SLAH1 is expressed in the root and plays a role in modulating chloride ion root to shoot transport [78]. SLAH2 is a nitrate-specific anion channel localized in plant roots that is impermeable to chloride ions. When a point mutation is introduced, SLAH2 turns into a nitrate/chloride anion channel [79]. During drought, a signaling cascade causes the activation of the SLAC1 anion channel via phosphorylation and stomatal closure. Upon phosphorylation, SLAC1 releases anions (e.g., Cl^−^ and NO_3_^−^) from the guard cells and depolarizes membrane potential, leading to the activation of K^+^_out_. Activated K^+^_out_ releases potassium ions along with water from the cell, thereby decreasing guard cell turgor pressure and closing down the stomatal pore.

**Figure 3:**
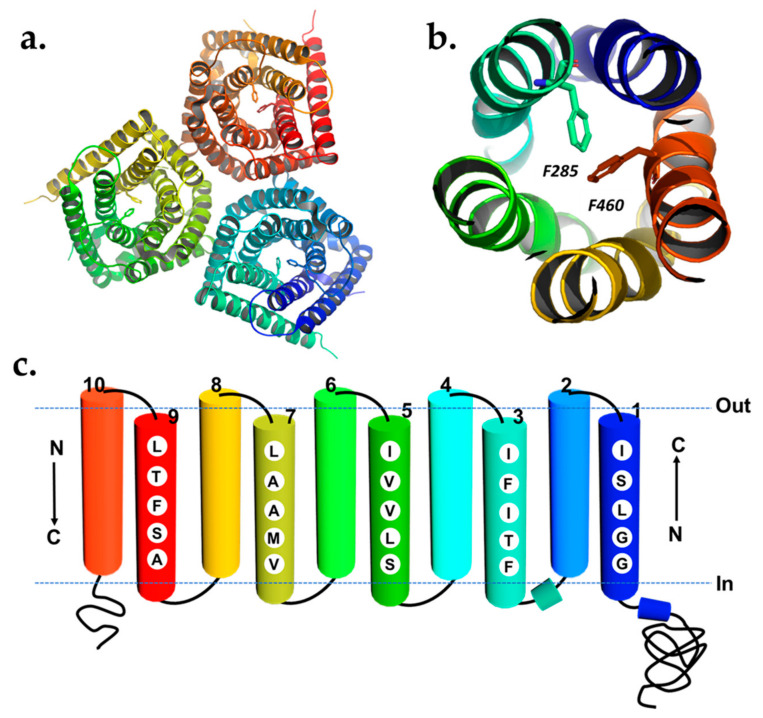
(**a**) The ribbon diagram of a *Bd*SLAC1 trimer (top view) colored spectrally. (**b**) *Bd*SLAC1 monomer is colored spectrally (top view) and the phenylalanine gating residues (F460 and F285) are shown in stick bonds within the channel pore. The pore-forming TM_odd_ helices are shown as a ribbon with TM_even_ removed. (**c**) Topology of SLAC1 subunit based on its structural model. Pore-lining residues are indicated in TM_odd_ helices of the *Bd*SLAC1 rolled-open model. (**a**,**b**) Were adopted from the structure, PDB entry code: 7EN0 [80], and generated using PyMol [66].

Recently, the cryo-EM structure of the *Bd*SLAC1 (SLAC1 anion channel from *Brachypodium distachyon*) was revealed [80]. The structure showed that *Bd*SLAC1 is a symmetric trimer and has a positive electrostatic potential surface on both the extracellular and cytoplasmic sides (Figure 3a). Each SLAC1 protomer has ten TM helices, arranged as five pairs of helical hairpins that are assembled into a unique protein fold with quasi-five-fold symmetry. TM_odd_ helices form a channel pore across the membrane, which is blocked by a highly conserved two phenylalanine residue-motif (F460 and F285 in *Bd*SLAC1/F450 and F276 in *At*SLAC1 (*Arabidopsis thaliana* SLAC1)). TM_even_ helices are straight and elongated but more inclined surrounding the inner pore while forming an external layer at the same time (Figure 3b,c).

SLAC1 has a unique structure and distinctive mechanism for gating. Dual high-energy phenyl rings of two conserved pore-lining phenylalanine residues interact and occlude the channel pore, and the channel vibrates between open and closed conformation. A recent study showed that there are six phosphorylation sites at the SLAC1 cytosolic N-terminus which can be phosphorylated by OST1 (Open stomata 1) kinase [80]. These multiple phosphorylation sites may act as a platform for other kinases to fine-tune and modulate SLAC1 activities. Highly conserved, positively charged residues on the cytoplasmic side provide potential interacting sites for the phosphorylated serine/threonine upon kinase activation. This interaction prompts the twisting of the pore-forming helices to unlatch the high-energy gate from occluding the pore as shown in Figure 3 [80,81].

In *Arabidopsis thaliana*, ALMT12 (also referred to as QUAC1; quick-activating anion channel 1) of the aluminum-activated malate transporter (ALMT) family, is predominately expressed in the guard cell and identified as the major component related to the R-type anion channel [16]. In response to several stimuli such as ABA, calcium, and CO_2_, stomatal closure was partly defective in *atalmt12* mutant plants [16,26,82]. This finding suggests that the guard cells might have other unidentified members of the R-type channels (QUACs). ALMT12 is permeable to malate, nitrate, and chloride ions and is activated through depolarization and inactivated by hyperpolarization rather than kinases [83]. Unlike *At*ALMT1, the activity of *At*ALMT12 is not governed by Al^3+^ [16,84]. Recently, the structural basis of the ALMT12 channel was revealed to be a symmetrical dimer that forms a pore with a T-shape which is responsible for passing anions across the membrane [85]. The ALMT12 structure is composed of two layers that can be divided into the TM and CH (cytoplasmic helical) domains. The TM domain consists of 6 V-shaped TM helices and the CH domain comprises 7 helices. Interestingly, there is a region in the CH domain enriched with serine/threonine residues that may contain a phosphorylation site for ALMT12 regulation [85].

In the guard cell tonoplast, there are three members of the ALMT family; *At*ALMT4, 6, and 9 [71,86,87,88,89]. *At*ALMT6 can mediate malate and fumarate fluxes depending on the tonoplast polarization state; upon tonoplast depolarization, a malate efflux occurs, while a malate influx occurs upon hyperpolarization. *At*ALMT6 is a Ca^2+^-activated channel and its activity is modulated by vacuole p^H^. *Atalmt6* mutant plants exhibited almost no phenotypic differences; however, the malate currents were reduced in the guard cell vacuole compared with wild-type plants [86]. Ye et al. showed that ALMT6 is involved in stomatal opening and the stomata opening was defective in ALMT6 in response to blue light [90]. On the other hand, *At*ALMT9 is activated by cytosolic malate and acts as a chloride efflux channel. An *atalmt9* mutant revealed that *At*ALMT9 plays a role in stomatal opening [87]. Another member of the tonoplast ALMT family member is *At*ALMT4, which can mediate anion fluxes in a phosphorylation-dependent manner. The study demonstrated the assembly of *At*ALMT4 in ABA-triggered stomatal closure. *Atalmt4* mutant plants exhibited impairment during ABA-triggered stomatal closure [91]. Malate influx through the guard cell PM is mediated by an ABC transporter (i.e., *At*ABCB14), and involved in stomatal opening [92]. Recently, a study showed that cytosolic malate can regulate stomatal movement in *Arabidopsis* via the indirect activation of S-type anion channels and such activity was abolished in OST1 and *cpk5/6/11/23* quadruple mutant guard cells [93]. The above-mentioned ALMTs illustrate the significant role of malate during stomatal movement. 

## 4. Guard Cell Signaling Elements Involved in Stress-Induced Stomatal Closure

ABA plays a main role in controlling stomatal closure via the activation of a complex signaling cascade that is mediated by different signaling elements including ABA receptors, kinase/phosphatases, and ion channels [94,95,96]. There are three kinase families involved in signaling pathways related to drought, osmotic stress, salt stress, and potassium deficiency [97,98,99]; the Sucrose Non-Fermenting 1 (SNF1) related protein Kinase-2 (SnRK2) family [100], the calcium-dependent protein kinase (CDPK/CPK) family [101], and the SnRK3 family (SNF1-related protein kinase 3)or CIPK; calcineurin B-like-interacting protein kinase [102]. In *Arabidopsis*, there are ten SnRK2s, among them, OST1/SnRK2.6 is a key regulator of the ABA-dependent stomatal closure [100]. OST1 is an ABA-dependent, calcium-independent protein kinase, whereas CPKs and CIPKs are calcium-dependent protein kinases that rely on calcium for their activity [102,103]. There are 34 CPK, 26 CIPK, and 10 CBLs members in *Arabidopsis*; CBL acts as a calcium sensor, not as a kinase. After calcium binding, CBL is activated and interacts with a kinase family CIPK leading to CIPK autophosphorylation and regulation of downstream target proteins including transcriptional factors, NADPH oxidases, transporters, and ion channels [104,105,106,107,108]. CIPKs are comprised of a kinase domain, an autoinhibitory (NAF/FISL) domain, and a phosphatase-binding domain (protein phosphatase interaction (PPI)). CIPK auto-inhibition is eliminated when the calcium-dependent CBL interacts with the NAF domain of CIPK [109]. As for CPKs, they have a calcium sensor domain and a kinase domain combined in one protein. CPKs have a C-terminal calmodulin-like domain that contains EF-hand motifs, which can directly bind to calcium. At low calcium concentration, kinases are autoinhibited; however, calcium is elevated during the response to different stimuli and initiates an interaction with EF-hands and changes its conformation that relieves the kinase auto-inhibition [103]. CPKs’ sensitivity to calcium varies greatly, with some CPKs such as CPK23 found to be insensitive to calcium [110,111]. CIB/CIPKs and CPKs share many targets for protein phosphorylation [109,112,113] (Table 1).

ABA receptor families of pyrabactin resistance 1/pyrabactin resistance 1-like (PYR/PYL) or receptor component of ABA receptor (RCAR) intracellularly perceive ABA and subsequently interact with PP2Cs clade A family (protein phosphatases of group C), resulting in phosphatase inactivation. In the absence of ABA, PP2Cs phosphatases dephosphorylate and inactivate SnRK2s, CIPK, and CPK families [24,111,114,115,116]. Under drought conditions, ABA, accumulated in the guard cell, is perceived by ABA receptors PYR/PYL, leading to PP2C inactivation and setting the different kinases free, such as OST1 to phosphorylate downstream targets and translate ABA signals into the appropriate cellular responses [117,118,119]. As for phosphatases, PP2Cs are a common target for calcium-dependent and calcium-independent kinases [120]. These kinase-phosphatase pairs play crucial roles in guard cell potassium channels and anion channel regulation (Table 1).

**Table 1:** Signaling elements involved in the regulation of guard cell stress response stomatal closure.

Kinases/Phosphatase/Ions	Protein Family	Target Channels	Localization	Channel Family	Ion Flux	Effect on Channel Activity	Reference
OST1	SnRK2	KAT1	PM	Shaker	K^+^ influx	Deactivation	[121,122]
		SLAC1	PM	SLAC/SLAH	Cl^−^ efflux	Activation	[117,123]
		*At*ALMT12	PM	ALMT	Malate efflux	Activation	[124]
CIPK5	SnRK3	GORK	PM	Shaker	K^+^ efflux	Activation	[125]
CIPK6	SnRK3	AKT1	PM	Shaker	K^+^ influx	Activation	[114,115]
		AKT2	PM	Shaker	K^+^ influx	Activation	[126]
CIPK23	SnRK3	AKT1	PM	Shaker	K^+^ influx	Activation	[114,127,128]
		SLAC1	PM	SLAC/SLAH	Cl^−^ efflux	Activation	[74]
		SLAH3	PM	SLAC/SLAH	Cl^−^ efflux	Activation	[74]
		CHL1	PM	NRT1	NO_3_ influx	Deactivation	[129]
		AtCLCa	Tonoplast	CLC	NO_3_ influx	Activation	[130,131,132]
CBL1	CBL	AKT1	PM	Shaker	K^+^ influx	Activation	[133]
CBL4	CBL	AKT1	PM	Shaker	K^+^ influx	Activation	[133]
CBL9	CBL	AKT1	PM	Shaker	K^+^ influx	Activation	[133]
CPK3	CPK/CDPK	KAT1/KAT2	PM	Shaker	K^+^ influx	Activation	[134]
		SLAC1	PM	SLAC/SLAH	Cl^−^ efflux	Activation	[116]
		GORK	PM	Shaker	K^+^ efflux	Activation	[134]
		TPK1	Tonoplast	TPK	K^+^ influx	Activation	[135]
CPK6	CPK/CDPK	GORK	PM	Shaker	K^+^ efflux	Activation	[134]
		SLAC1	PM	SLAC/SLAH	Cl^−^ efflux	Activation	[111,136,137]
CPK13	CPK/CDPK	KAT1/KAT2	PM	Shaker	K^+^ influx	Activation	[138]
		GORK	PM	Shaker	K^+^ efflux	Activation	[134]
CPK21	CPK/CDPK	GORK	PM	Shaker	K^+^ efflux	Activation	[139]
		SLAC1	PM	SLAC/SLAH	Cl^−^ efflux	Activation	[111]
		SLAH3	PM	SLAC/SLAH	Cl^−^ efflux	Activation	[24]
CPK33	CPK/CDPK	GORK	PM	Shaker	K^+^ efflux	Activation	[134]
ABI1	Clade A PP2C	SLAC1	PM	SLAC/SLAH	Cl^−^ efflux	Deactivation	[140]
ABI2	Clade APP2Cs	GORK	PM	Shaker	K^+^ efflux	Deactivation	[141]
AtPP2CA	Clade APP2Cs	AKT2	PM	Shaker	K^+^ influx	Deactivation	[142,143]
		GORK	PM	Shaker	K^+^ efflux	Deactivation	[141]
		SLAC1	PM	SLAC/SLAH	Cl^−^ efflux	Deactivation	[117]
AIP1	Clade APP2Cs	AKT1	PM	Shaker	K^+^ influx	Deactivation	[114,115]
Calcium	-	AtALMT4	Tonoplast	ALMT	Malate fluxes	Activation	[91]
		AtALMT6	Tonoplast	ALMT	Malate fluxes	Activation	[86]
Cytosolic malate	-	AtALMT9	Tonoplast	ALMT	Cl^−^ efflux	Activation	[87]

While ABA-mediated stomatal closure is considered to be the core abiotic stress signaling pathway, there is evidence that it is intertwined with multiple other signaling pathways, including the calcium pathway [120,144]. In the guard cell, cytosolic calcium acts as a second messenger to regulate ion channels, primarily by activating S-type anion channels and down-regulation of K^+^_in_ channels, thus inhibiting stomatal opening and mediating stomatal closure [145,146]. In plants, stress signals that increase cytosolic ABA levels also elevate cytosolic calcium signals, and several protein targets that are regulated in response to abiotic stimuli are regulated by both ABA and calcium-mediated signaling [22,120,147]. The SLAC1 and KAT1 channels-coordinated activity are clear examples of how ABA-mediated and calcium-mediated signaling are interconnected in response to environmental stimuli.

## 5. Signaling Mechanisms in Guard Cell during Stress-Induced Stomatal Closure 

**Figure 4:**
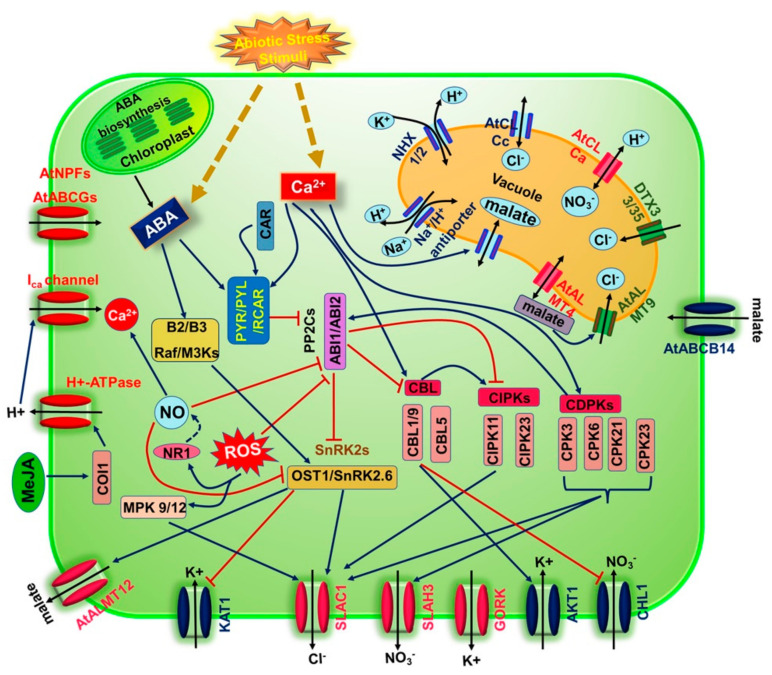
The schematic diagram represents the regulation of ion channels involved in the stomatal movement induced by stress in *Arabidopsis thaliana*. Channels that participate in stomatal closing or opening are colored in pink or blue, respectively. The arrows through the channels are ion effluxes/influx (black); straight arrows (blue), and broken arrows (red) are activation and inhibition respectively. Channels colored in green; the role they play during stomatal movement still needs further investigation.

During drought, the anion effluxes and the associated potassium release from the guard cells reduce the guard cell turgor pressure and lead to stomatal closure. The ABA hormone regulates such guard cell activities through the coordinated activation and inhibition of the PM anion and cation channels. ABA levels in guard cells rise due to de novo biosynthesis, recycling inactive conjugates (such as ABA glycose ester; ABA-GE) via β-glucosidases BG1 and BG2, and import, while they decline as a result of hydroxylation, conjugation, and export [148,149,150]. During plant water stress in *Arabidopsis*, nitrate transporter 1/peptide transporter family (NPF) member NPF4.6, and ABCG40 the ATP-binding cassette transporter are ABA importers, whereas DTX50 (from the multidrug and toxin efflux (MATE) transporter family) and AtABCG25 are ABA exporters [151,152,153,154] (for review, see References [155,156,157,158]). It has been suggested that rapid stomatal response under abiotic stress may depend mainly on ABA synthesized in the guard cell (autonomous synthesis in the guard cell), whereas in the long term soil water shortage, ABA synthesized in the vascular may play a significant role [148,158]. In the guard cell, increased cytosolic ABA hormone activates both S- and R-type anion channels [159,160]. The core of the ABA signaling cascade is the activation of the key OST1 kinase, which is achieved when cytosolic ABA binds to ABA receptors (PYR/PYL/RCAR) and the protein phosphatase ABI1, subsequently resulting in ABI1 inactivation [100,118,161,162]. Activated OST1 phosphorylates the N-terminus of S-type anion channel SLAC1, which results in unlatching the phenyl ring that occludes the channel pore and thus activates the channel [80,81,117,123,163]. *Ost1* mutants show low S-type anion channel activity and were unable to induce guard cell stomatal closure [123]. OST1 can also phosphorylate and inactivate KAT1 to reduce the potassium influx to the guard cell, which asserts its significance in stomatal closure [121,122]. In addition, ABA-activated OST1 can phosphorylate NADPH oxidase RbohF and RbohD, resulting in reactive oxygen species (ROS) being produced in the guard cell leading to the stomatal closure [121,163,164]. Two more kinases, mitogen-activated protein kinase 9 (MPK9) and MPK12, can induce ROS-mediated S-type anion channel activation in the guard cell during ABA signaling [165,166]. Upon ROS production, ABI2 not ABI1 was inhibited, suggesting its indirect role in enhancing the activity of GHR1 [167,168,169]. Furthermore, ROS generation in the guard cell stimulates nitrate reductase 1 (NR1) to produce more nitric oxide (NO), which can be used to regulate stomatal movement [170,171,172,173,174]. Receptor-like plasma membrane kinase guard cell hydrogen peroxide resistant 1 (GRH1), was also identified as an ABA-dependent regulator for SLAC1 activation. Unlike OST1, GHR1 interacts with ABI2 phosphatase rather than ABI1 and may work in tandem with OST1 to close stomata in response to ABA [168]. A recent study showed that SLAC1 activation by GHR requires the interaction of calcium dependent protein kinase CPK3 with GHR1 and that GHR1 has a scaffold function in stomatal closure [175]. In ABA-induced stomatal closure, brassinosteroid insensitive 1-associated receptor kinase 1 (BAK1) formed a complex with OST1 in response to ABA *in planta* that enhances stomatal closure. BAK1-OST1 complex formation is inhibited by ABI1 signifying the importance of ABI1 in OST1 regulation [176]. A recent study showed BAK1-flagellin sensing 2 (FLS2) complex, physically interacts with stress-induced factor 2 (SIF2) receptor like-kinase and phosphorylate and activates SLAC1 which induces stomatal closure upon bacterial invasion. In addition, the *sif2-1* mutant was defective in response to ABA, signifying SIF2 importance in ABA-mediated stomatal closure (Figure 4) [177]. 

Recently, several members of the Raf/M3Ks (MAPKK-kinase family) that can regulate SnRK2 kinases in the ABA signaling pathway were identified [178,179,180,181]. These studies have revealed novel roles for the B2/B3 family of Raf/M3Ks in modulating the ABA signaling pathway via phosphorylation of SnRK2s from subclass III, and for the B4 family of Raf/M3Ks kinases in modulating the ABA-independent signaling pathway via phosphorylation of SnRK2s from subclass I under osmotic stress [178,179,180,181,182] (for review, see reference [183]). For instance, Takahashi et al., identified members of the M3Ks (MAPKK-kinase family) that can activate SnRK2 kinases in response to ABA. The study showed M3Ks can phospho-activate SnRK2s and SLAC1 activation *in planta* in an ABA-dependent manner and the plants with triple M3K knockout have impaired ABA stress response [179]. On the contrary, *Arabidopsis* C family members of Raf/M3Ks (Raf36 and Raf22) negatively regulate ABA signaling and under abiotic stress, SnRK2 phosphorylate and promote Raf36 degradation [184]. 

On the other hand, ABA increases cytosolic calcium concentrations that can activate S-and R-type anion channels and inhibit proton pumps causing plasma membrane depolarization, which leads to K^+^_in_ channels inhibition and K^+^_out_ activation [43,67,68,69,185,186]. The increase in cytosolic calcium concentration is achieved by the calcium influx into the guard cell through calcium transporters and calcium release from its intracellular stores [21,68,187,188,189,190,191]. It is known that ABA induces the production of ROS that increases cytosolic calcium concentrations via the activation of calcium-permeable I_Ca_ channels [147,169,192,193]. CBL1/CBL9 is activated when calcium levels rise, forming a complex with CIPK26 and phosphorylating RbohF, increasing ROS production via a positive feedback loop [194,195]. Similarly, calcium activates CPK4, 5, 6, and 11 kinases that phosphorylate RbohD positively, hence regulating ROS production [196,197]. Additionally, the accumulated ROS promotes NO synthesis, which in turn releases calcium from intracellular calcium stores [173,198,199]. The increase in calcium concentration by these different mechanisms activates different CPKs in the guard cell, leading to phosphorylation and activation of the key SLAC1 anion channel. The kinases that activate SLAC1 are CIPK11/CBL5, CIPK23/CBL1or CBL9, and CPK3, 6, 21, 23 [74,111,116,140,200]. Although these kinases activate SLAC1 in *Xenopus* oocytes, they phosphorylate different residues from OST1 (for example, CPK phosphorylates S59 at the SLAC1 N-terminus) [74,140]. Several studies showed the presence of sophisticated cross-talk between SLAC1-regulating kinases, as both calcium-dependent protein kinases and OST1 activity are inhibited by ABI1and ABI2 [74,111,113,140]. CPK sensitivity for calcium activation varies between these family members. For instance, CPK21 is highly sensitive to calcium, whereas CPK23 is weakly sensitive to calcium [111]. However, SLAC1 activation *in planta* appears to be much more complex regarding both kinases’ dependence. *Arabidopsis* plant lines with impaired function of triple mutants SnRK2S (*snrk2.2/2.3/2.6*) or CPK quadruple mutant plants (*cpk5/6/11/23*) show no SLAC1 activity and no stomatal closure in response to calcium or ABA [137]. According to Huang et al., most of the Ca^2+^ signals are elicited during the acceleration phase of stomatal closure, which is triggered by OST1 suggesting that these signals will boost stomatal closure via enhancing the S-anion channel activity [144]. These findings indicate that SLAC1 boosted activation required a joined function of CPKs and SnRK2s. Additionally, CBL5-CIPK11 and CBL1/9-CIPK23 showed SLAC1 activity in oocytes and CBL1/9 showed an impaired stomatal response (Figure 4) [74,114,200]. 

ABA-induced S-type anion channel activity was disrupted when *cpk3cpk6* or *cpk5cpk6cpk11cpk23* mutations were introduced [136,137], while *in planta*, these mutants showed impaired stomatal closure upon ABA induction. There are some confusing results reported from calcium-dependent protein kinases activity experiments and that might be due to the functional overlap between these kinases. Even though CPK21 activation by 14-3-3 proteins activates the K^+^_out_ channel, GORK, and coordinates with SLAC1 for stomatal closure, the *cpk21* mutant showed greater tolerance to osmotic stress [139,201,202]. SLAH3 along with SLAC1 anion channel activities are required for stomatal closure [203,204]. Unlike SLAC1, SLAH3 cannot be activated by calcium-independent OST1 kinase [24]. However, the calcium-dependent protein kinases that activate SLAC1 can also activate SLAH3 such as CIPK23-CBL1/CBL9, CPK3, CPK6, CPK21, and CPK23 [24,74,75]. Interestingly, Zhang et al. reported that SLAC1 and SLAH3 can interact and inactivate KAT1 [72]. The nitrate transporter CHL1 is expressed in the guard cell and participates in nitrate build-up in the guard cell and contributes to stomatal opening. Similar to SLAH3, CHL1 is phosphorylated by CIPK23-CBL1/CBL9, reducing the nitrate intake (negatively regulated) and may have crosstalk between them [129,205,206,207]. 

In *Arabidopsis*, the ALMT12 channel is considered the main constituent of the R-type anion channels in the guard cell [16,26,82]. When OST1 and ALMT12 are co-expressed in *Xenopus* oocytes, there is a marked R-type anion channel activation indicating that OST1 plays a role in R-type anion channel activation [124]. In *Arabidopsis*, *slac1*, *slah3*, or *almt12* single mutant retained some stomatal response to ABA, reduced air humidity, and elevated CO_2_, whereas *slac1*-*almt12* double mutant has almost no response to these stimuli, signifying the importance of S and R-type anion channels coordinated activation in stomatal closure [208]. The *At*ALMT12 C-terminus, located at the cytosol, acts as a sensor and the channel is inactivated when the membrane potential is hyperpolarized [83]. A Patch-clamp study showed that malate-dependent ALMT12 activity is dependent on cytosolic calcium and the channel current increased with the increasing cytosolic calcium concentration. The study further shows that the channel is co-regulated by calmodulin indicating a complex regulation mechanism for ALMT12 [209].

The GORK channel represents the major voltage-gated K^+^_out_ channel in the guard cell and the absence of GORK activity impaired stomatal closure [25,210]. The PM depolarization activates GORK to induce potassium efflux; however, Ooi et al. reported that the direct interaction between ABA and GORK enhances the potassium efflux through the GORK channel and such a mechanism can represent a rapid stomatal response to environmental stimuli, rather than the conventional ABA signaling [211]. Nonetheless, Förster et al. showed that wound-triggered jasmonic acid (JA) hormone signaling can phospho-activate the GORK channel through the CBL1-CIPK5 kinase complex in coordination with the ABA signaling cascade [125]. Furthermore, the GORK channel may operate as ligand-gated channels as they possess binding domains that enable them to be modulated by different molecules such as G-proteins, inositol, protein phosphatases, ATP, cyclic nucleotides, and gamma-aminobutyric acid under environmental stresses (Figure 4) [212]. 

Although little is known about ions released from the vacuoles, potassium fluxes across the tonoplast play an essential role in controlling stomatal movement. The TPK1 vacuole potassium channel appears to be the main player in this process. Upon calcium elevation in the cytosol, TPK1 tonoplast channel activation by CPK3 plays a role in stomatal closure [135,213]. Furthermore, in response to ABA, Kinase 7 (KIN7), a receptor-like kinase, phosphorylates and activates the TPK1 channel, resulting in stomatal closure [214,215]. Taken together, the drought hormone ABA is perceived in the guard cell cytoplasm by ABA-receptors, which might act as a signal cascade that forks into calcium-independent and calcium-dependent branches [216,217]. Calcium-independent OST1 kinase activates the S-and R-type anion channels in the PM and mediates the release of anions from the guard cell. On the other hand, the elevated ABA causes an elevation in cytosolic calcium through calcium uptake as well as calcium release during a process regulated by ROS and nitric oxide [167,173,218,219], which in turn leads to S-and R-type anion channel activation and PM depolarization [220]. The depolarized PM inactivates K^+^_in_ channels and triggers the activation of the K^+^_out_ channel to induce potassium efflux. The released ions drive water out of the guard cells lowering turgor pressure and causing stomatal closure [25,210,221,222].

## 6. Conclusions

Understanding how the guard cell adapts to different environmental stimuli and deciphering the underlying signaling mechanisms and structural aspects will provide an insight into guard cell response modulated by different stimuli as well as offers knowledge of signal transduction in plants. Stomatal movement in response to abiotic stress such as drought is regulated via a complex signaling mechanism that involves different guard cell ion channels and their regulatory signaling components, such as hormones, calcium, protein kinases, NO, ROS, and receptors. In this mini-review, we described the recent advances in understanding how coordinated activities of the ion channels in the guard cell provide a defensive mechanism for plants to cope with different environmental stress conditions. Despite our tremendous knowledge of the guard cell signaling cascades, and their involvement in plant adaptation to abiotic stress, gaps in our knowledge remain and many questions remain unanswered, such as how SLAC1 structure conformation changes upon phosphorylation, the structural basis for the anion channels’ anion selectivity, the guard cell calcium channel molecular aspects and their connection to calcium signaling pathways and the oligomerization of the guard cell channels and the role it plays in the regulation of plants’ abiotic stress. Lately use of Cryogenic electron microscopy has been a revelation in identifying the structure of several plants’ ion channels and can be a key to solving the molecular structures of many unidentified plant ion channels in the near future. Integrated studies of structural insights into these different ion channels at the molecular level, as well as studies of their physiological mechanisms, might broaden our understanding of signaling mechanisms and signal transduction during various abiotic stresses. Such comprehension and information will aid in engineering plants with improved responses to drought, higher CO_2_ levels, and other environmental stresses.

## Data Availability

Not applicable.
